# Hemodynamic Definitions, Phenotypes, Pathophysiology, and Evaluation of Pulmonary Hypertension Related to Left Heart Disease

**DOI:** 10.3390/jcdd12070238

**Published:** 2025-06-22

**Authors:** Elizabeth C. Ghandakly, Akshat Banga, Roop Kaw

**Affiliations:** 1 Department of Internal Medicine, Cleveland Clinic, 9500 Euclid Avenue, Cleveland, OH 44195, USA; ghandae@ccf.org; 2 Department of Internal Medicine, Mount Auburn Hospital, Harvard Medical School, Cambridge, MA 02138, USA; akshat.banga@mah.harvard.edu; 3 Departments of Hospital Medicine and Outcomes Research Anesthesiology, Cleveland Clinic, 9500 Euclid Avenue, Cleveland, OH 44195, USA

**Keywords:** pulmonary hypertension, left heart disease

## Abstract

Pulmonary hypertension (PH) can develop from multiple etiologic mechanisms and disease states. Of all such conditions, left-sided heart disease (LHD) is commonly understood to be the most common etiology or mechanism. Given the widespread prevalence of left heart disease and the prognostic implications of PH, early diagnosis is imperative. More recently, the diagnostic cut-offs for mean pulmonary arterial pressure as well as peripheral vascular resistance have been lowered to achieve this objective. Despite these revised standards, the current indications for right heart catheterization are mostly aimed at identifying advanced disease. Proven vasodilator therapies for pulmonary arterial hypertension have so far not shown a meaningful role in the management of PH in LHD. This is largely related to the fact that multiple mechanisms and co-morbidities can independently lead to the development of PH in an individual patient. Understanding and identifying those phenotypes remain important in devising future treatment strategies. Molecular pathways that eventually lead to irreversibility of PH can provide another frontier in the pharmacologic management of PH in LHD.

## 1. Hemodynamic Definitions

### 1.1. Hemodynamic Basics

#### 1.1.1. Historical Context

Reports of pulmonary hypertension (PH) as a disease date back to the 1800s, based on anecdotal symptoms and autopsy findings [1]. In 2009, Kovacs et al. published a systematic review of right heart catheterization (RHC) data, indicating that the normal mean arterial pulmonary artery pressure (mPAP) for a healthy patient at rest was 14.0 ± 3.3 mmHg [2]. The 2018 European Society of Cardiology/European Respiratory Society guidelines for the diagnosis and treatment of PH thus formally decreased the PH threshold to two standard deviations above the mPAP of this standard for a normal patient at rest, and the pulmonary vascular resistance (PVR) threshold from 3 Wood units (WU) to 2 WU [3].

#### 1.1.2. Hemodynamic Definitions of Pulmonary Hypertension

Graph 2. PH is the most common subset and refers to PH that is due to left-sided heart disease (LHD) [4]. PH due to LHD is classified by mPAP ≥ 20 mmHg and a pulmonary capillary wedge pressure (PCWP) above 15 mmHg [3]. The currently accepted hemodynamic definitions of PH are made with respect to RHC findings and are described in Table 1 below.

It is important to consider the value of PCWP in the context of a pretest probability of heart failure with preserved ejection fraction (HFpEF), since up to 20% of patients with normal PCWP can be misdiagnosed as having pulmonary arterial hypertension (PAH) [5]. If the pretest probability is high based on the number of risk factors for HFpEF, provocative testing with intravenous fluid challenge or exercise should be considered even at a recorded PCWP of 13–15 mmHg. If a fluid challenge of 500 cc of normal saline (or 7 cc/kg) over 5 min raises the PCWP immediately to 18 mmHg, PH-LHD is likely [6,7]. At PCWP > 15 mmHg, left ventricular end diastolic pressure (LVEDP) should be validated [8].

Further characterization of PH has undergone modifications based on the threshold for PVR based on several studies that relate survival to hemodynamics [9,10,11]. According to the 2022 European Cardiology Society/European Respiratory Society guidelines for PH, if the PVR is <2 Wood units, this is referred to as isolated post-capillary PH (ipc-PH) and is believed to be related to passive transmission of elevated filling pressures in the pulmonary circulation. Combined post-capillary pulmonary hypertension (Cpc-PH) occurs when active remodeling of pulmonary vasculature causes the PVR to exceed 2 Wood units [3].

The diastolic pulmonary gradient (DPG) and trans-pulmonary gradient (TPG) are measurements that can further assess pulmonary vascular hemodynamics [12]. The DPG is the gradient between diastolic PA pressure and mean PCWP, whereas the TPG is the gradient between mean PA pressure and left atrial (LA) pressure. Together, these have historically been used in diagnosing PH and differentiating between pre- and post-capillary PH. A low DPG (typically less than 7 mmHg) has been said to suggest IpcPH, and a high TPG (typically greater than 12 mmHg) to suggest involvement of the pulmonary vasculature (high PVR, low pulmonary vascular compliance, and pronounced response to exercise) and, thus, CpcPH [13]. Even at similar levels of PCWP, patients with HFpEF have higher TPG and PVR than other patients. This reflects more severe pulmonary vascular disease under conditions of similar pulsatile loading [14].

### 1.2. Pulmonary Vascular Resistance vs. Pulmonary Vascular Compliance

The hemodynamic definition of PVR takes into account only the static resistive load, while in reality, the pulmonary arterial blood flow is pulsatile. Increased PCWP can determine the right ventricular (RV) afterload also by decreasing the pulsatile component or Pulmonary artery compliance (PAC) to accommodate the blood ejected by the RV during the systole [15]. PAC is defined as the change in volume produced by a given change in pressure. (Delta V/Delta P), commonly estimated as a ratio between RV stroke volume and PA pulse pressure. PAC refers to how well the pulmonary arteries distend in systole and return to baseline in diastole. In this way, it is a measure of the elasticity and distensibility of the vessels throughout the pulmonary vasculature. Unlike the systemic circulation, most of this is contributed by the arterial bed distal to the main pulmonary arteries (about 85%) [16].

Because PVR is calculated by subtracting PCWP from mPAP, all over cardiac output, a lower PVR indicates elevated left atrial (LA) pressure without intrinsic pathology at the level of the pulmonary vasculature [13]. In contrast to the systemic circulation, the relationship between PVR and PVC is inverse and hyperbolic [17]. Also, changes in PAC precede those of PVR. This relationship can result in two types of changed volume: one that can fill a cylindrical vessel and make it round, reflecting higher pulsatile RV load without any increased pressure (or PVR in this case), referred to as the unstressed volume [18,19], and one that is accompanied by a change in pressure, referred to as the stressed volume [20]. Early stages of this so-called “arteriolization”, which may cause collagen deposition and luminal narrowing of the pulmonary veins, can be reversed by mitigating the LA pressure [21].

## 2. Pathophysiology

### 2.1. Role of Systemic Co-Morbidities in PH-LHD

Unlike PAH, which primarily arises from intrinsic pulmonary vascular disease, PH-LHD is profoundly influenced by systemic co-morbidities, including obesity, diabetes, chronic kidney disease (CKD), and hypertension, characterized as metabolic syndrome. These conditions promote systemic endothelial dysfunction, oxidative stress, and metabolic dysregulation, all of which contribute to microvascular dysfunction, pulmonary arterial stiffening, and right heart failure (RHF) [13,22]. Obesity and metabolic syndrome are being increasingly recognized as key drivers of PH-LHD, with mechanistic studies demonstrating adipose tissue-derived inflammation, leptin-induced vasoconstriction, and insulin resistance as contributors to vascular remodeling [23]. Adipokine dysregulation and chronic low-grade inflammation disrupt nitric oxide (NO) signaling and enhance endothelin (ET)-1 expression, leading to increased pulmonary vascular tone and fibrosis [24,25]. A direct association has been reported between a high-fat diet in a mouse model as well as ectopic adipose tissue accumulation and the severity of PH-HFpEF, noting increases in right ventricular pressure (RVSP), LVEDP, and bi-ventricular hypertrophy [26].

In addition to endothelial dysfunction, dysregulated glucose metabolism contributes to maladaptive vascular remodeling. Hyperglycemia promotes advanced glycation end-product (AGE) accumulation, which stimulates oxidative stress and inflammation in the pulmonary vasculature. This results in excessive extracellular matrix (ECM) deposition and arterial stiffening, compounding the progression of PH-LHD [27]. In fact, in Ranchoux’s mouse model, mice with both metS and diastolic dysfunction increased expression of IL-6, which induced a proinflammatory state leading to high PVR that was then reversed with metformin [26]. CKD represents another systemic amplifier of PH-HFpEF, with elevated levels of fibroblast growth factor-23 (FGF-23), oxidative stress, and vascular calcification contributing to endothelial dysfunction and increased PVR [28,29].

### 2.2. Molecular Pathways in PH-HFpEF

At the cellular and molecular level, PH-LHD involves a complex interplay of endothelial dysfunction, inflammatory changes, metabolic dysregulation, and maladaptive vascular signaling pathways. Among these, the nitric oxide (NO)-cyclic guanosine monophosphate (cGMP), ET-1, transforming growth factor-beta (TGF-β), and mitogen-activated protein kinase (MAPK) pathways play pivotal roles.

Dysregulated NO-cGMP signaling is a hallmark of PH-LHD. Chronic inflammation triggers endothelial dysfunction, which reduces NO bioavailability, leading to decreased activation of soluble guanylate cyclase (sGC) and cGMP production [30]. This, in turn, impairs vasodilation and promotes vascular smooth muscle cell (VSMC) and fibroblast proliferation, leading to excess extracellular matrix (ECM) deposition and pulmonary arterial fibrosis [31]. Concurrently, the upregulation of ET-1 exacerbates vasoconstriction, vascular hypertrophy, and fibrosis, closely mirroring mechanisms observed in PAH [32].

Elevated levels of interleukin (IL)-6, tumor necrosis factor (TNF)-α, C-reactive protein (CRP), and other pro-inflammatory cytokines correlate with increased pulmonary vascular stiffness and RH dysfunction [27]. Inflammatory signaling pathways such as nuclear factor (NF)-κB and JAK-STAT also contribute to the activation of pulmonary VSMCs, leading to pathological remodeling and heightened vascular tone. Additionally, monocyte and macrophage infiltration in pulmonary arterioles induces further fibrosis and vascular narrowing, worsening PH [33].

Emerging evidence also suggests that lipid metabolism may play a critical role in PH-HFpEF progression. Recent studies indicate that dysregulated omega-3 fatty acid metabolism and increased glycolytic activity in pulmonary vascular cells promote vascular proliferation and inflammation, suggesting a potential metabolic target for future therapies [27,32]. Furthermore, recent studies have implicated the fatty acid binding protein 5 (FABP5) and signal transducer and activator of transcription 3 (STAT3) pathways as additional molecular culprits driving PH-LHD progression. FABP5 is associated with lipid metabolism dysregulation and pulmonary vascular inflammation, while STAT3 promotes inflammatory signaling and enhances vascular fibrosis [26,34].

### 2.3. Dysregulation of Cardiovascular Remodeling

Cardiovascular remodeling in PH-LHD is a culmination of mechanical stress, neurohormonal activation, and molecular derangements. Central to this process is the persistent activation of TGF-β signaling, which orchestrates fibroblast proliferation and collagen deposition, leading to pulmonary arterial stiffening and maladaptive RV remodeling [35,36]. Simultaneously, BMP pathway suppression removes a critical brake on this fibrotic cascade, facilitating progressive vascular and myocardial remodeling, contributing to endothelial dysfunction and perivascular fibrosis, exacerbating pulmonary vascular resistance and RV afterload [37,38]. Oxidative stress and reactive oxygen species (ROS) further exacerbate vascular injury. ROS generation activates the NF-κB pathway, promotes endothelial-mesenchymal transition (EndoMT), and facilitates ECM accumulation, compounding pulmonary vascular resistance and right heart strain [39,40,41]. Moreover, mitochondrial dysfunction induced by chronic hypoxia, metabolic syndrome, and inflammation impairs cardiomyocyte energetics, leading to maladaptive hypertrophy and eventual RV failure [42]. This is further aggravated by EndoMT, a process where endothelial cells acquire mesenchymal phenotypes, exacerbating extracellular matrix deposition and vascular fibrosis, impairing PAC [43,44]. Chronic neurohormonal activation via the renin-angiotensin-aldosterone system (RAAS) further exacerbates vascular remodeling by promoting vasoconstriction, salt retention, and fibrosis. Angiotensin-II enhances TGF-β signaling and oxidative stress, while aldosterone exacerbates myocardial fibrosis and vascular stiffening [45,46].

### 2.4. Hemodynamic and Structural Pathogenesis of PH-LHD

Unlike IpcPH, which is predominantly a hydrostatic disorder, CpcPH exhibits features of active pulmonary vascular disease, including vasoconstriction, medial hypertrophy, endothelial dysfunction, adventitial fibrosis, perivascular inflammation, and increased extracellular matrix deposition [40,47]. Chronic pulmonary venous congestion and capillary stress failure further contribute to microvascular dysfunction, exacerbating pulmonary arterial pressures even in the absence of overt left ventricular systolic dysfunction [48,49,50]. Unlike in IpcPH, CpcPH shows features resembling PAH, including increased ET-1, reduced NO bioavailability, and impaired prostacyclin signaling [51]. These changes further contribute to an increased TPG and PVR, reinforcing the notion that CpcPH represents an advanced and distinct pathophysiologic phenotype of PH-LHD [52].

### 2.5. Right Ventricular Dysfunction and Ventricular-Arterial Uncoupling

While the LV remains the primary driver of PH in LHD, the adaptation and subsequent decompensation of the RV are key factors influencing disease progression and prognosis. In the early stages of PH-LHD, the RV initially compensates for increased afterload by undergoing adaptive hypertrophy and increased contractility, characterized as right ventricular (RV)-pulmonary artery (PA) coupling. As the PVR rises in CpcPH, the RV undergoes progressive dilation, fibrosis, ischemia, and contractile dysfunction, ultimately leading to RV-PA uncoupling and RV dysfunction [53]. With RV-PA uncoupling, RV end-diastolic pressures (EDP) rise, and RV function deterioration ensues, characterized by a decline in RV longitudinal strain, reduced tricuspid annular plane systolic excursion (TAPSE), and tricuspid regurgitation, finally resulting in RA pressure elevation and systemic venous congestion [54,55,56]. The hemodynamic consequences of RV dysfunction extend beyond the pulmonary circulation, as septal bowing into the LV impairs diastolic filling, further deteriorating LVEF, reducing cardiac output in a phenomenon known as ventricular interdependence, which is a key hallmark of advanced PH-LHD [57,58]. The resultant reduction in LV preload and stroke volume exacerbates systemic hypotension and exercise intolerance, contributing to worsening clinical outcomes.

Although PH-LHD and PAH share common mechanistic features, RV failure in PH-LHD occurs in the setting of elevated left-sided filling pressures, increased pulmonary venous congestion, and diastolic dysfunction [38]. In PH-LHD, RV dysfunction is often more insidious, making therapeutic interventions particularly challenging [59]. This unique hemodynamic profile is distinct from PAH, where RV dysfunction primarily arises from intrinsic pulmonary vascular disease.

## 3. Evaluation of PH-LHD

### 3.1. Right Heart Catheterization

Right heart catheterization (RHC) is the gold standard for diagnosing PH, providing direct measurements of mPAP, PCWP, CO, and PVR [60]. However, inaccuracies in hemodynamic measurement can lead to misclassification of PH and therefore should be looked at in the overall context of the clinical picture, non-invasive studies, as well as dynamic PCWP response to IV fluids or exercise. Wherever LHD is dominant clinically and PH appears to be mild, RHC is not required [40].

Attention to detail must be paid when obtaining the PCWP measurement itself. The inaccurate placement of the PA catheter can lead to misleading numbers and, thus, create the potential for misdiagnosis. The correct placement of the PA catheter tip is in the distal pulmonary artery. If the PA catheter tip is placed too proximally in the main PA artery, the PCWP will be underestimated because the balloon may not completely occlude blood flow (under-wedging) [61]. It should be inflated in the RA and then advanced to the correct position. Proper PA catheter placement should be strictly followed, which includes correctly placing the transducer. It should be set to zero at the mid-thoracic line. If pre-capillary PH is suspected, and PCWP is >15 mmHg or PCWP tracings are atypical, a PCWP oxygen hemoglobin saturation should be obtained, and if lower than 90–95%, the PA catheter should be repositioned [62].

The measurement of PCWP can be affected a great deal by respiratory swings affecting thoracic pressure, which are minimized at the end of expiration. While ESC/ERS recommends end-expiratory measurement of PCWP, the issue remains controversial, as the potential misclassification of 29% of precapillary PH was reported as post-capillary PH [63]. PCWP measured at the end expiratory cycle corresponds to functional residual volume, which represents equilibration of intra- and extra-thoracic pressures. The mean of three measurements should be recorded at end-expiration rather than at the mid-respiratory cycle. In morbidly obese patients, intra-thoracic fluctuations are wide, which can lead to underestimation of PCWP [64,65].

### 3.2. Limitations of PCWP, MPAP, and PVR

The availability of RHC is often limited to specialized centers, leading to underutilization and delayed diagnosis in many patients with suspected PH-LHD [13]. RHC is also invasive and associated with procedural risks, particularly in elderly or frail patients [66]. In addition, the accurate measurement of PCWP, especially in patients with HFpEF or atrial fibrillation, can be technically challenging and subject to inter-operator variability. Both under-wedging and over-wedging have also been reported to falsely elevate PCWP [65,67]. It is important to consider the value of PCWP in the context of a pretest probability of HFpEF, since up to 20% of patients with normal PCWP can be misdiagnosed as having PAH [5].

There are limitations to consider with respect to the mPAP and the PVR as well. The absolute value of mPAP does not matter so much as the clinical context, and sole reliance on mPAP, especially if the number is borderline, such as 21–24 mmHg, can be misleading [68,69]. MPAP as an absolute number is not sufficient by itself in determining the severity of and outcomes from PH as long as the RV adapts to the afterload. The uncoupling of the RV to pulmonary circulation results in increased RV filling pressures/dilation/failure, right atrial pressure (RAP) > 15 mmHg, and low CO, all of which will eventually decrease exercise capacity. Although the severity of PH is often determined on the basis of hemodynamics, mPAP is not consistently prognostic, even though it will increase initially with progression of PVR. It will, however, start falling as the RV failure sets in [70]. Clinical context, such as WHO functional capacity and 6 min walk distance (MWD), should be taken into account when reading an absolute mPAP number. MPAP may be elevated from other, non-pulmonary causes such as left-to-right cardiac shunting or increased cardiac output [69].

Similarly, PVR, a holy grail of diagnosis, is also not without its limitations. The 6th World Symposium on Pulmonary Hypertension emphasized the importance of PVR in diagnosis by including the 3 WU and under threshold to safeguard against over-diagnosis, but some have noted that the clinical context and trajectory of patients must be taken into account [71]. PA compliance should be taken into consideration along with PVR because it is sensitive to elasticity and pulsatility, and thus may better reflect elevated RV load in some circumstances [72]. Because there is an inverse relationship between PAC and PVR, a gray zone exists where PVR may remain normal or rise only slightly while PA compliance begins to fall, thus affecting RV afterload. This effect on RV afterload contributes to a greater incidence of right heart failure and a less favorable prognosis [38]. Finally, PVR can be misinterpreted in patients with left-to-right shunting, such as in the case of congenital heart disease or in cases of chronic thromboembolism or severe chronic obstructive pulmonary disease (COPD) [69].

## 4. Echocardiography

While RHC is the gold standard for diagnosing PH, echocardiography is the first-line screening tool for screening and early detection and is widely available. A resting TR velocity ≥ 2.9 is highly suggestive of PH. Recent studies have developed variables based on sPAP to estimate RV after load scores that can help identify and differentiate increased resistive and pulsatile RV load, and patients with high RV after load scores were found to be more likely to have CpcPH [73]. Echocardiography can also provide insights into predominant phenotypes [6].

Some major limitations of echocardiography are the underestimation of TR velocity due to the truncation of continuous wave Doppler in patients with annular dilatation and tethering from functional TR [74], the possibility that the TR jet may be absent even in the presence of severe PH, and the fact that TR estimates may be low in patients with COPD [75]. Additional sources of error in the measurement of sPAP can be inaccurate estimates of RAP [76], arrhythmias, and the use of diuretics [77].

### Semi-Recumbent Bicycle Stress Echocardiography

While it is not currently recommended for screening, semi-recumbent bicycle echocardiography [78] can help differentiate pre- and post-capillary phenotypes. An exertional pulmonary artery systolic pressure (PASP) increase associated with worsening of LV diastolic parameters is suggestive of a post-capillary phenotype, while no increase in the latter parameters suggests a pre-capillary phenotype [79]. Worsening rest-stress mitral E/e′ > 12 and worsening MR and or/TR + high RVSP with exercise also suggest a post-capillary phenotype [80].

## 5. Pulmonary Vascular Phenotypes

### 5.1. Why May Phenotypes Be Needed?

Compared to other groups of PH, there has been little progress, if any, in the approach to clinical management of PH-LHD. Conventionally, the management of underlying heart disease has been emphasized, but the very presence of PH complicates that approach. Along similar lines, the management of HFpEF is equally complex due to the complexity of underlying pathophysiologic models. On top of this classic indications for RHC, like eligibility for cardiac transplant, chronic thromboembolic pulmonary hypertension (CTEPH), etc., create a referral bias, and as a result, only the more severe spectrum of PH-LHD is represented.

PH can develop heterogeneously by many mechanisms in the same patient, and oftentimes, a patient may belong to more than one PH group across groups 1–5. Conceptually, previously described categories of PH etiology are heterogeneous. The American Thoracic Society (ATS) has stated that the existing classification system of PH has not led to the customization of treatment based on patient characteristics and responsiveness [81]. The ATS has therefore noted that far greater than five subtypes of PH exist in the broader patient population and, thus, methods of understanding and describing this heterogeneity are crucial in order to individualize care. The National Heart, Lung, and Blood Institute developed a prospective cohort for studying and phenotyping patients with PH called PVDOMICS (Redefining Pulmonary Hypertension through Pulmonary Vascular Disease Phenomics) [82,83,84]. While lung structure and function are more impaired in Group 3 PH, it is common to have pulmonary abnormalities in Group 2 PH, even if it is determined to be purely isolated left-heart-disease-driven PH [85]. These include spirometric abnormalities and obstructive lung disease on pulmonary function testing. This is not entirely surprising, as the PARAGON-HF trial has shown that 14% of patients with HFpEF also carry a diagnosis of COPD, and those with COPD had higher New York Heart Association (NYHA) classifications. Abnormal spirometry is itself independently associated with an increased risk of HFpEF [86].

### 5.2. Heterogeneity Within Type 2 PH

A typical case of PH-LHD may represent more than one phenotype of PH, and these may respond to treatment differently and even in contrast with a particular therapy. This is true with respect to Group 2 PH, as various causes of left-sided heart disease can each lead to PH presentations. Clinical evidence has shown that certain combinations of underlying disease, combined with resultant PH, will lead to worse clinical presentations and outcomes.

PH-LHD broadly includes patients with HFpEF, those who are less likely to have PH (younger patients with normal brain-natriuretic peptide), those with elevated pulmonary pressures (often with coexisting obesity and diabetes), and those who are more likely to have PH (patients with co-morbid ischemic heart disease and sometimes CKD) [87]. Using a cluster analysis, after strictly excluding HFpEF (even potential HFpEF), pulmonary function abnormalities/lung disease, including veno-occlusive disease, CKD in a homogenous PAH cohort, and PCWP, when followed longitudinally for 10 years, showed evolution into group 2 PH physiology in those with PCWP > 12 mmHg at baseline, 25% of patients resembling a left heart PAH phenotype [88]. In this way, certain co-morbidities or specifics of a patient’s clinical presentation can aid in phenotyping patients within the Type 2 PH classification and, ideally, lead to more nuanced and targeted therapies among those phenotypes.

More importantly, HFpEF can be easily misdiagnosed with WHO group 1 PAH. Recent studies have also shown that patients with group 1 PH with risk factors for and higher pretest probability for HFpEF, as assessed by HFpEF-ABA score, can have a marked PCWP dynamic response to NO, exercise, and fluid challenge and a poor response to vasodilator therapy [89,90]. Since HFpEF is more prevalent than group 1 PH, and diagnosis often requires unmasking by provocative hemodynamic maneuvers on RHC, a high probability of HFpEF was uncovered in this study of patients with group 1 PH [5,13,90,91].

As noted above, another study suggests a continuum between idiopathic pulmonary arterial hypertension and Type 2 PH associated with HFpEF [92]. The presence of certain clinical criteria, specifically three or more of the following: ischemic heart disease, diabetes, hypertension, or obesity, suggests shared features along that continuum that include PH related to LHD. A subpopulation has been reported between classic IPAH and PH-LHD with a severe pre-capillary component. Some phenotypes within PH due to LHD demonstrate more severe pulmonary vasculopathy, which is associated with a worse prognosis. The molecular mechanisms that lead to certain phenotypic presentations are incompletely understood, but they drive some patients with PH due to LHD to transition from functional PH requiring only volume correction to a phenotype that is resistant to volume correction. This type of vascular remodeling that occurs in some patients and not others further contributes to how best to approach various phenotypic presentations therapeutically [14]. The CpcPH phenotype discussed earlier, with elevated PVR and decreased PA compliance, has been found to be associated with an exonic single-nucleotide polymorphism in certain cellular pathways—something not seen in patients with the IpcPH phenotype [38]. Similarly, patients with HFrEF are more likely to have eccentric hypertrophy and intrinsic pulmonary vascular remodeling with RV pathology, which can lead to earlier RV failure through ventricular-arterial uncoupling, as opposed to patients with HFpEF with metabolic pathology that lead to LA stiffness.

### 5.3. Tests to Aid Determination of Phenotypes

As phenotypes continue to be identified and better defined, some tests have emerged to aid these classifications. An exercise RHC is one mainstay. One study referred to older adults with suspected PH and risk factors for PH due to LHD to undergo exercise RHC [93]. In this cohort, the exercise RHC led to a significant reclassification of patients from Type 1 PAH to PH due to LHD. Overall, the hemodynamic classification by using exercise RHC changed from 36% no PH, 44% Type 1 PH, and 20% PH due to LHD to 15% no PH, 36% Type 1 PH, and 49% PH due to LHD. Such higher-sensitivity tests can aid proper diagnosis and, thus, guide more appropriate treatment based on phenotypes.

Notably, a phenotype known as exercise-induced pulmonary hypertension has been observed in patients with HFpEF. In this phenomenon, diastolic ventricular interaction occurs during exercise—specifically in obese patients with HFpEF. This leads to restricted left ventricular filling by the right ventricle and thus a phenotype of PH due to LAD is unmasked by exercise. There are some important diagnostic measurements that can significantly aid in phenotyping patients with PH due to LHD [94]. Table 2 provides a summary of some relevant tests.

## 6. Current Evidence for Pharmacologic Treatment of PH-LHD

The role of pulmonary vasodilators approved for PAH remains controversial in the treatment of PH-LHD. Randomized trials have shown limited efficacy and potential harm with endothelin receptor antagonists (ERAs), phosphodiesterase-5 inhibitors (PDE5i), and soluble guanylate cyclase (sGC) stimulators in this population.

In regard to guideline-directed medical therapy (GDMT), recent randomized controlled trial (RCT) data on SGLT2 inhibitors make a strong case for use in HFpEF-PH [95,96,97]. Emerging RCT data have shown improvement in PASP and mPAP by improving vascular endothelial function (decreased ET1) and inflammation (decreased CRP, IL6, and TNF-α) with Sacubitril-Valsartan + Dapagliflozin [98]. The recommendation to use Sacubitril-Valsartan alone in HFpEF-PH is based on limited data [99,100]. Table 3 provides a summary of some recent trials.

## 7. Prognostic Significance of Hemodynamic Factors in PH-LHD

The presence of PH does carry a worse prognosis in patients with LHD as compared to those without PH. PSAP ≥ 48 mmHg in patients diagnosed with HFpEF has been reported as an independent predictor of mortality [13]. Conflicting data regarding DPG as a prognostic indicator have been observed. In patients with LHD-PH, those with DPG 7 mmHg or higher had shorter periods of survival [113] and higher rates of mortality [114]. But such differentiation in DPG has not been shown to predict mortality in patients with idiopathic cardiomyopathy [115]. It has been theorized that this may be due to DPG’s role in predicting pulmonary vascular remodeling in patients with CpcPH [116]. With respect to TPG, studies have largely shown that it has a limited role in prognosis with respect to mortality [115].

PVR ≥ 3 WU is associated with higher mortality [115,117]. PVR has also been shown to be a superior prognostic indicator to both DPG and TPG, as CpcPH has a worse prognosis overall [118]. Ultimately, PH associated with HFrEF has higher mortality and rates of cardiac hospitalization than PH associated with HFpEF, and a PVR of 3 WU or higher and TPG of 12 mmHg or higher indicate higher mortality risk for patients with HFpEF [119]. While PA compliance is not currently used for prognosis, it is an emerging potential predictor of mortality associated with LHD-driven PH [120]. In one study of HFpEF patients, PA compliance less than 1.1 mL/mmHg had a higher sensitivity in mortality prediction than PVR, DPG, and TPG [121]. There are, however, no defined thresholds or reference values for PA compliance, and additional studies are still needed for more granular prognostication.

Right ventricular dysfunction has also been shown to have an association with both mortality and hospital readmission. In one study of patients with HFpEF, right ventricular dysfunction defined through assessment of tricuspid annular plane systolic excursion of 55 mmHg or greater had higher PA systolic pressures and higher mortality and readmission hazard [122]. Finally, studies have also examined potential molecular prognostic indicators. One such indicator is plasma ET-1. One study of patients with HFrEF found elevated levels of this molecule to be an independent predictor of one-year mortality [123].

## Figures and Tables

**Table 1:** Hemodynamic definitions of Type 2 Pulmonary Hypertension.

Definition	Hemodynamics	Clinical Groups
Pulmonary Hypertension	mPAP > 20 mmHg	Disease Categories/States
Precapillary PH	mPAP > 20 mmHg PVR > 2 WU PCWP < 15 mmHg	Pulmonary arterial hypertension PH due to lung disease CTEPH
Combined precapillary + postcapillary PH (CpcPH)	mPAP > 20 mmHg PVR > 3 WU PCWP > 15 mmHg PCWP/LVEDP > 15 mmHg *TPG > 12* mmHg *DPG > 7* mmHg	Left-sided heart disease Left heart disease + lung disease overlap
Isolated postcapillary PH	mPAP > 20 mmHg PVR < 3 WU PCWP/LVEDP > 15 mmHg *TPG < 12* mmHg *DPG < 7* mmHg	Left-sided heart disease
Exercise PH	mPAP/CO slope between rest and exercise > 3 mmHg/L per min	Exertional dyspnea with preserved ejection fraction and normal resting PCWP

mPAP: mean pulmonary arterial pressure; PVR: pulmonary vascular resistance; PCWP: pulmonary capillary wedge pressure; TPG: transpulmonary gradient; DPG: diastolic pulmonary gradient; CTEPH: chronic thromboembolic pulmonary hypertension; LVEDP: Left ventricular end diastolic pressure; CO: cardiac output.

**Table 2:** Summary of the relevant tests in Phenotyping of type 2 Pulmonary Hypertension.

Diagnostic Test	Importance for Phenotypic Determination
Right heart catheterization (RHC)	RV and RA pressures showing RV-PA coupling
RHC with CMR, SPECT, or 3D Echo	Can add examination of RV volume
Exercise RHC	Slope of mPAP/CO showing hemodynamic response to exercise Increase in PCWP > 25 mmHg with exercise
RHC with volume challenge	Hemodynamic response to volume overload
Semi-recumbent exercise bicycle echocardiography	Worsening rest-stress mitral E/e′ > 12 and worsening MR and/or TR + high RVSP in response to exercise (suggest post-capillary PH)
Cardiac MR	RV strain to show RV-PA coupling
Lung ultrasound Chest CT	Congestion score—shows pulmonary congestion Extravascular lung water (EVLW) assessment
Pulmonary CT angiography/VQ scan	Perfusion defects and ventilation-perfusion mismatch
Pulmonary HRCT	Pulmonary interstitial congestion
Pulmonary function tests (PFTs)	DLCO shows pulmonary vascular remodeling and pulmonary congestion Decreased FVC suggests Type 3 PH
Exhaled breath analysis	Mass spectrometry of exhaled breath can show volatile compounds indicating heart failure
CardioMEMs	Implantable PAP sensors showing PAP, PA changing in different settings over time PA pressure response to exercise
Phenomapping with machine learning	Phenotyping of PH-LHD

**Table 3:** Summary of recent trials on treatment of Type 2 Pulmonary Hypertension.

Trial/Study	Author	Year	Intervention	Design	Population	Key Findings	Conclusion
SERENADE	Shah et al. [101]	2025	Macitentan (ERA)	RCT, n = 142	HFpEF/HFmrEF (≥40%) and pulmonary vascular disease: PVR ≥ 3 WU or PH + RV dysfunction	No improvement in NTproBNP or clinical status; higher rate of fluid retention and cardiac events.	Macitentan did not improve outcomes and showed potential harm; ERAs should be avoided in PH-HFpEF.
Sacubitril-Valsartan + Dapagliflozin for PH-LHD	Ge T et al. [98]	2023	Sacubitril-Valsartan (ARNI) + Dapagliflozin (SGLT2i)	RCT, n = 120	PH due to left heart disease (mPAP ≥ 25 mmHg, PAWP > 15 mmHg)	Combination therapy significantly improved LVEF, 6MWD, mPAP, PASP, endothelial function (↑ NO, ↓ ET-1), NT-proBNP + inflammatory markers vs. sac/val alone. No increase in ADRs.	Sacubitril-Valsartan+ Dapagliflozin safely improves cardiac function, vascular health, and inflammation in PH-LHD.
SilHF	Cooper et al. [102]	2022	Sildenafil (PDE5i)	RCT, n = 69	HFrEF + PASP ≥ 40 mmHg	No improvement in symptoms, QoL, PASP, or 6MWT vs. placebo.	Sildenafil was safe but ineffective in PH-HFrEF.
HELP Trial	Burkhoff et al. [103]	2021	Levosimendan (Ca sensitizer)	RCT, n = 37	PH-HFpEF (LVEF ≥ 40% and NYHA class II–III, mPAP ≥ 35 mmHg, and baseline PCWP ≥ 20 mmHg)	No significant reduction in PCWP at peak exercise, but reduced PCWP across all stages and improved 6MWD.	Levosimendan improved hemodynamics and exercise tolerance in PH-HFpEF, but warrants further investigation due to limited participants.
EMBRACE-HF	Nassif et al. [97]	2021	Empagliflozin (SGLT2i)	RCT, n = 65	HF (EF 44%), CardioMEMS sensor	↓ PA diastolic pressure by 1.7 mmHg; no change in QoL or NT-proBNP.	Empagliflozin reduces PA pressures in HF regardless of EF.
VICTORIA	Armstrong et al. [104]	2020	Vericiguat (sGC stimulator)	RCT, n = 5050	HFrEF with recent hospitalization or IV diuretic use	Modest but significant reduction in primary composite outcome of cardiovascular death or first HF hospitalization.	Vericiguat modestly reduced HF hospitalization and CV death in high-risk HFrEF patients.
CAPACITY HFpEF	Udelson et al. [105]	2020	Praliciguat (sGC stimulator)	RCT, n = 181	HFpEF (EF ≥ 40%), NO-deficiency phenotype	No significant change in peak VO_2_, 6MWT, or ventilatory efficiency.	Praliciguat did not improve exercise capacity or symptoms in HFpEF.
PADN-5	Zhang et al. [106]	2019	PADN (Pulmonary Artery Denervation) vs. Sildenafil + sham	RCT, n = 128	PH-LHD (mPAP ≥ 25 mmHg, PCWP > 15 mmHg, PVR > 3 WU)	PADN improved 6MWD, NT-proBNP, mPAP, and PVR vs. Sildenafil group.	PADN was superior to Sildenafil for improving functional and hemodynamic status in PH-LHD.
MELODY-1	Vachiery et al. [107]	2018	Macitentan (ERA)	RCT, n = 63	CpcPH with LVEF ≥ 30%, PVR ≥ 3 WU	No benefit; higher fluid retention and adverse events.	Not recommended for PH-LHD. Fluid overload risk evident early in treatment.
SIOVAC	Bermejo et al. [48]	2021	Sildenafil (PDE5i)	RCT, n = 200	PH after successful valve surgery	Sildenafil arm had worse clinical outcomes; increased clinical events and reduced 6MWD.	Contraindicated in post-VHD PH-LHD.
BADDHY Trial	Koller et al. [108]	2017	Bosentan (ERAs)	RCT, n = 20	PH-HFpEF and RV dysfunction	No improvement in 6 MWD or pulmonary pressure in Bosentan group; better outcomes in placebo group; trial. terminated early.	Bosentan offered no benefit and may be harmful. in PH-HFpEF.
Effects of Sildenafil on Invasive Hemodynamics and Exercise Capacity in HFpEF and PH	Hoendermis et al. [109]	2015	Sildenafil (PDE5i)	RCT, n = 52	PH-HFpEF (LVEF ≥ 45% and NYHA class II–IV)	No significant change in pulmonary hemodynamics or exercise capacity.	Sildenafil did not significantly improve outcomes in PH-HFpEF patients.
LEPHT	Bonderman et al. [110]	2013	Riociguat (sGC stimulator)	RCT, n = 201	HFrEF with mPAP ≥ 25 mmHg and LVEF ≤ 40%	Primary endpoint (mPAP reduction) not met. However, significant improvement in CI, PVR, SVR, and QoL.	Promising hemodynamic signal but insufficient for routine use.
Sildenafil Improves Exercise Capacity and QoL in Patients With Systolic Heart Failure and Secondary PH	Lewis et al. [111]	2007	Sildenafil (PDE5i)	RCT, n = 34	HFrEF + PH	Sildenafil significantly improved peak VO_2_, reduced PVR, improved 6MWT, QoL, and RVEF; no change in PAP on RHC.	Sildenafil improved exercise capacity, hemodynamics, and QoL in HFrEF with PH.
FIRST	Califf et al. [112]	1997	Epoprostenol (Prostacyclin)	RCT, n = 471	Advanced HF (LVEF < 25%)	↑ CI and ↓ PCWP, but ↑ mortality, no QoL or walk distance benefit.	Stopped early due to harm; not effective for PH-LHD.

## Data Availability

No new data were created or analyzed in this study.

## References

[1] Hewes J.L., Lee J.Y., Fagan K.A., Bauer N.N. (2020). The changing face of pulmonary hypertension diagnosis: A historical perspective on the influence of diagnostics and biomarkers. Pulm. Circ..

[2] Kovacs G., Berghold A., Scheidl S., Olschewski H. (2009). Pulmonary arterial pressure during rest and exercise in healthy subjects: A systematic review. Eur. Respir. J..

[3] Humbert M., Kovacs G., Hoeper M.M., Badagliacca R., Berger R.M.F., Brida M., Carlsen J., Coats A.J.S., Escribano-Subias P., Ferrari P. (2022). 2022 ESC/ERS Guidelines for the diagnosis and treatment of pulmonary hypertension. Eur. Heart J..

[4] Maron B.A. (2023). Revised Definition of Pulmonary Hypertension and Approach to Management: A Clinical Primer. J. Am. Heart Assoc..

[5] Robbins I.M., Hemnes A.R., Pugh M.E., Brittain E.L., Zhao D.X., Piana R.N., Fong P.P., Newman J.H. (2014). High Prevalence of Occult Pulmonary Venous Hypertension Revealed by Fluid Challenge in Pulmonary Hypertension. Circ. Heart Fail..

[6] D’Alto M., Badesch D., Bossone E., Borlaug B.A., Brittain E., Humbert M., Naeije R. (2021). A Fluid Challenge Test for the Diagnosis of Occult Heart Failure. Chest.

[7] Fujimoto N., Borlaug B.A., Lewis G.D., Hastings J.L., Shafer K.M., Bhella P.S., Carrick-Ranson G., Levine B.D. (2013). Hemodynamic Responses to Rapid Saline Loading. Circulation.

[8] Inampudi C., Silverman D., Simon M.A., Leary P.J., Sharma K., Houston B.A., Vachiéry J.-L., Haddad F., Tedford R.J. (2021). Pulmonary Hypertension in the Context of Heart Failure with Preserved Ejection Fraction. Chest.

[9] Kolte D., Lakshmanan S., Jankowich M.D., Brittain E.L., Maron B.A., Choudhary G. (2018). Mild Pulmonary Hypertension Is Associated with Increased Mortality: A Systematic Review and Meta-Analysis. J. Am. Heart Assoc..

[10] Maron B.A., Brittain E.L., Hess E., Waldo S.W., Barón A.E., Huang S., Goldstein R.H., Assad T., Wertheim B.M., Alba G.A. (2020). Pulmonary vascular resistance and clinical outcomes in patients with pulmonary hypertension: A retrospective cohort study. Lancet Respir. Med..

[11] Maron B.A., Hess E., Maddox T.M., Opotowsky A.R., Tedford R.J., Lahm T., Joynt K.E., Kass D.J., Stephens T., Stanislawski M.A. (2016). Association of Borderline Pulmonary Hypertension with Mortality and Hospitalization in a Large Patient Cohort: Insights From the Veterans Affairs Clinical Assessment, Reporting, and Tracking Program. Circulation.

[12] Peled Y., Beigel R., Ram E., Klempfner R., Lavee J., Patel J., Raanani E. (2021). What is High Risk? Hemodynamic Definitions of Pulmonary Hypertension and Heart Transplantation Outcomes: A Contemporary Cohort Analysis. J. Heart Lung Transplant..

[13] Al-Omary M.S., Sugito S., Boyle A.J., Sverdlov A.L., Collins N.J. (2020). Pulmonary Hypertension Due to Left Heart Disease. Hypertension.

[14] Adir Y., Guazzi M., Offer A., Temporelli P.L., Cannito A., Ghio S. (2017). Pulmonary hemodynamics in heart failure patients with reduced or preserved ejection fraction and pulmonary hypertension: Similarities and disparities. Am. Heart J..

[15] Tedford R.J., Hassoun P.M., Mathai S.C., Girgis R.E., Russell S.D., Thiemann D.R., Cingolani O.H., Mudd J.O., Borlaug B.A., Redfield M.M. (2012). Pulmonary Capillary Wedge Pressure Augments Right Ventricular Pulsatile Loading. Circulation.

[16] Saouti N., Westerhof N., Helderman F., Tim Marcus J., Boonstra A., Postmus P.E., Vonk-Noordegraaf A. (2010). Right Ventricular Oscillatory Power Is a Constant Fraction of Total Power Irrespective of Pulmonary Artery Pressure. Am. J. Respir. Crit. Care Med..

[17] Vonk Noordegraaf A., Westerhof B.E., Westerhof N. (2017). The Relationship Between the Right Ventricle and its Load in Pulmonary Hypertension. J. Am. Coll. Cardiol..

[18] Dragu R., Rispler S., Habib M., Sholy H., Hammerman H., Galie N., Aronson D. (2015). Pulmonary arterial capacitance in patients with heart failure and reactive pulmonary hypertension. Eur. J. Heart Fail..

[19] Lankhaar J.-W., Westerhof N., Faes T.J.C., Tji-Joong Gan C., Marques K.M., Boonstra A., van den Berg F.G., Postmus P.E., Vonk-Noordegraaf A. (2008). Pulmonary vascular resistance and compliance stay inversely related during treatment of pulmonary hypertension. Eur. Heart J..

[20] Magder S., Malhotra A., Hibbert K., Hardin C. (2021). Cardiopulmonary Monitoring Basic Physiology, Tools, and Bedside Management for the Critically Ill: Basic Physiology, Tools, and Bedside Management for the Critically Ill.

[21] Fayyaz A.U., Edwards W.D., Maleszewski J.J., Konik E.A., Dubrock H.M., Borlaug B.A., Frantz R.P., Jenkins S.M., Redfield M.M. (2018). Global Pulmonary Vascular Remodeling in Pulmonary Hypertension Associated with Heart Failure and Preserved or Reduced Ejection Fraction. Circulation.

[22] Vachiéry J.-L., Tedford R.J., Rosenkranz S., Palazzini M., Lang I., Guazzi M., Coghlan G., Chazova I., De Marco T. (2019). Pulmonary hypertension due to left heart disease. Eur. Respir. J..

[23] Shi X.-F., Su Y.-C. (2020). Vascular Metabolic Mechanisms of Pulmonary Hypertension. Curr. Med. Sci..

[24] Humbert M., Guignabert C., Bonnet S., Dorfmüller P., Klinger J.R., Nicolls M.R., Olschewski A.J., Pullamsetti S.S., Schermuly R.T., Stenmark K.R. (2019). Pathology and pathobiology of pulmonary hypertension: State of the art and research perspectives. Eur. Respir. J..

[25] Jia Q., Ouyang Y., Yang Y., Yao S., Chen X., Hu Z. (2023). Adipokines in pulmonary hypertension: Angels or demons?. Heliyon.

[26] Ranchoux B., Nadeau V., Bourgeois A., Provencher S., Tremblay É., Omura J., Coté N., Abu-Alhayja’a R., Dumais V., Nachbar R.T. (2019). Metabolic Syndrome Exacerbates Pulmonary Hypertension due to Left Heart Disease. Circ. Res..

[27] Aradhyula V., Vyas R., Dube P., Haller S.T., Gupta R., Maddipati K.R., Kennedy D.J., Khouri S.J. (2024). Novel insights into the pathobiology of pulmonary hypertension in heart failure with preserved ejection fraction. Am. J. Physiol.-Heart Circ. Physiol..

[28] Faul C., Amaral A.P., Oskouei B., Hu M.-C., Sloan A., Isakova T., Gutiérrez O.M., Aguillon-Prada R., Lincoln J., Hare J.M. (2011). FGF23 induces left ventricular hypertrophy. J. Clin. Investig..

[29] Widmann L., Keranov S., Jafari L., Liebetrau C., Keller T., Troidl C., Kriechbaum S., Voss S., Arsalan M., Richter M.J. (2023). Fibroblast growth factor 23 as a biomarker of right ventricular dysfunction in pulmonary hypertension. Clin. Res. Cardiol..

[30] Stasch J.-P., Evgenov O.V., Humbert M., Evgenov O.V., Stasch J.-P. (2013). Soluble Guanylate Cyclase Stimulators in Pulmonary Hypertension. Pharmacotherapy of Pulmonary Hypertension.

[31] Triposkiadis F., Xanthopoulos A., Skoularigis J., Starling R.C. (2022). Therapeutic augmentation of NO-sGC-cGMP signalling: Lessons learned from pulmonary arterial hypertension and heart failure. Heart Fail. Rev..

[32] Madonna R., Biondi F., Ghelardoni S., D’Alleva A., Quarta S., Massaro M. (2024). Pulmonary hypertension associated to left heart disease: Phenotypes and treatment. Eur. J. Intern. Med..

[33] Agrawal V., Kropski J.A., Gokey J.J., Kobeck E., Murphy M.B., Murray K.T., Fortune N.L., Moore C.S., Meoli D.F., Monahan K. (2023). Myeloid Cell Derived IL1β Contributes to Pulmonary Hypertension in HFpEF. Circ. Res..

[34] Lei Q., Yu Z., Li H., Cheng J., Wang Y. (2022). Fatty acid-binding protein 5 aggravates pulmonary artery fibrosis in pulmonary hypertension secondary to left heart disease via activating wnt/β-catenin pathway. J. Adv. Res..

[35] Allen B.J., Frye H., Ramanathan R., Caggiano L.R., Tabima D.M., Chesler N.C., Philip J.L. (2023). Biomechanical and Mechanobiological Drivers of the Transition From PostCapillary Pulmonary Hypertension to Combined Pre−/PostCapillary Pulmonary Hypertension. J. Am. Heart Assoc..

[36] Dituri F., Cossu C., Mancarella S., Giannelli G. (2019). The Interactivity between TGFβ and BMP Signaling in Organogenesis, Fibrosis, and Cancer. Cells.

[37] Maruyama H., Sakai S., Ieda M. (2022). Endothelin-1 alters BMP signaling to promote proliferation of pulmonary artery smooth muscle cells. Can. J. Physiol. Pharmacol..

[38] Weatherald J., Hemnes A.R., Maron B.A., Mielniczuk L.M., Gerges C., Price L.C., Hoeper M.M., Humbert M. (2024). Phenotypes in pulmonary hypertension. Eur. Respir. J..

[39] Alamri A.K., Ma C.L., Ryan J.J. (2022). Left Heart Disease-Related Pulmonary Hypertension. Cardiol. Clin..

[40] Rosenkranz S., Gibbs J.S.R., Wachter R., De Marco T., Vonk-Noordegraaf A., Vachiéry J.-L. (2016). Left ventricular heart failure and pulmonary hypertension. Eur. Heart J..

[41] Rosenkranz S., Lang I.M., Blindt R., Bonderman D., Bruch L., Diller G.P., Felgendreher R., Gerges C., Hohenforst-Schmidt W., Holt S. (2018). Pulmonary hypertension associated with left heart disease: Updated Recommendations of the Cologne Consensus Conference 2018. Int. J. Cardiol..

[42] Wang Y., Wang Y., Zhang W. (2025). Dysregulation of Mitochondrial in Pulmonary Hypertension-Related Right Ventricular Remodeling: Pathophysiological Features and Targeting Drugs. Rev. Cardiovasc. Med..

[43] Lu X., Gong J., Dennery P.A., Yao H. (2019). Endothelial-to-mesenchymal transition: Pathogenesis and therapeutic targets for chronic pulmonary and vascular diseases. Biochem. Pharmacol..

[44] Peng Q., Shan D., Cui K., Li K., Zhu B., Wu H., Wang B., Wong S., Norton V., Dong Y. (2022). The Role of Endothelial-to-Mesenchymal Transition in Cardiovascular Disease. Cells.

[45] Kaparianos A., Argyropoulou E. (2011). Local renin-angiotensin II systems, angiotensin-converting enzyme and its homologue ACE2: Their potential role in the pathogenesis of chronic obstructive pulmonary diseases, pulmonary hypertension and acute respiratory distress syndrome. Curr. Med. Chem..

[46] Kuba K., Imai Y., Penninger J. (2006). Angiotensin-converting enzyme 2 in lung diseases. Curr. Opin. Pharmacol..

[47] Naeije R., Gerges M., Vachiery J.-L., Caravita S., Gerges C., Lang I.M. (2017). Hemodynamic Phenotyping of Pulmonary Hypertension in Left Heart Failure. Circ. Heart Fail..

[48] Bermejo J., González-Mansilla A., Mombiela T., Fernández A.I., Martínez-Legazpi P., Yotti R., García-Orta R., Sánchez-Fernández P.L., Castaño M., Segovia-Cubero J. (2021). Persistent Pulmonary Hypertension in Corrected Valvular Heart Disease: Hemodynamic Insights and Long-Term Survival. J. Am. Heart Assoc..

[49] Omura J., Bonnet S., Kutty S. (2019). Right ventricular and pulmonary vascular changes in pulmonary hypertension associated with left heart disease. Am. J. Physiol.-Heart Circ. Physiol..

[50] Riley J.M., Fradin J.J., Russ D.H., Warner E.D., Brailovsky Y., Rajapreyar I. (2024). Post-Capillary Pulmonary Hypertension: Clinical Review. J. Clin. Med..

[51] Caravita S., Faini A., Carolino D’Araujo S., Dewachter C., Chomette L., Bondue A., Naeije R., Parati G., Vachiéry J.-L. (2018). Clinical phenotypes and outcomes of pulmonary hypertension due to left heart disease: Role of the pre-capillary component. PLoS ONE.

[52] Rezaee M.E., Nichols E.L., Sidhu M., Brown J.R. (2016). Combined Post- and Precapillary Pulmonary Hypertension in Patients with Heart Failure. Clin. Cardiol..

[53] Fauvel C., Damy T., Berthelot E., Bauer F., Eicher J.-C., De Groote P., Trochu J.-N., Picard F., Renard S., Bouvaist H. (2024). Post-capillary pulmonary hypertension in heart failure: Impact of current definition in the PH-HF multicentre study. Eur. Heart J..

[54] Calcutteea A., Lindqvist P., Soderberg S., Henein M.Y. (2014). Global and Regional Right Ventricular Dysfunction in Pulmonary Hypertension. Echocardiography.

[55] Lindqvist P., Henein M.Y. (2012). Right ventricular function in pulmonary hypertension. Imaging Med..

[56] Lv G., Li A., Zhai Y., Li L., Deng M., Lei J., Tao X., Gao Q., Xie W., Zhai Z. (2025). Assessment of right ventricle–to–pulmonary artery coupling by three-dimensional echocardiography in pre-capillary pulmonary hypertension: Comparison with tricuspid annular plane systolic excursion /systolic pulmonary artery pressure ratio. BMC Med. Imaging.

[57] Fang H., Wang J., Shi R., Li Y., Li X.M., Gao Y., Shen L.T., Qian W.L., Jiang L., Yang Z.G. (2024). Biventricular Dysfunction and Ventricular Interdependence in Patients with Pulmonary Hypertension: A 3.0-T Cardiac MRI Feature Tracking Study. J. Magn. Reson. Imaging.

[58] Pinsky M.R. (2016). The right ventricle: Interaction with the pulmonary circulation. Crit. Care.

[59] Ltaief Z., Yerly P., Liaudet L. (2023). Pulmonary Hypertension in Left Heart Diseases: Pathophysiology, Hemodynamic Assessment and Therapeutic Management. Int. J. Mol. Sci..

[60] McNeil K. (2008). Assessment of Patients with Pulmonary Hypertension. Respiration.

[61] Rosenkranz S., Preston I.R. (2015). Right heart catheterisation: Best practice and pitfalls in pulmonary hypertension. Eur. Respir. Rev..

[62] Viray M.C., Bonno E.L., Gabrielle N.D., Maron B.A., Atkins J., Amoroso N.S., Fernandes V.L.C., Maran A., Nielsen C.D., Powers E.R. (2020). Role of Pulmonary Artery Wedge Pressure Saturation During Right Heart Catheterization. Circ. Heart Fail..

[63] LeVarge B.L., Channick R.N. (2015). The changing paradigm in pulmonary hypertension trials: Longer duration, new endpoints. Curr. Opin. Pulm. Med..

[64] Boerrigter B.G., Waxman A.B., Westerhof N., Vonk-Noordegraaf A., Systrom D.M. (2014). Measuring central pulmonary pressures during exercise in COPD: How to cope with respiratory effects. Eur. Respir. J..

[65] Mathier M.A. (2011). The Nuts and Bolts of Interpreting Hemodynamics in Pulmonary Hypertension Associated with Diastolic Heart Failure. Adv. Pulm. Hypertens..

[66] Wattanachayakul P., Kittipibul V., Salah H.M., Yaku H., Gustafsson F., Baratto C., Caravita S., Fudim M. (2025). Invasive haemodynamic assessment in heart failure with preserved ejection fraction. ESC Heart Fail..

[67] Tonelli A.R., Mubarak K.K., Li N., Carrie R., Alnuaimat H. (2011). Effect of Balloon Inflation Volume on Pulmonary Artery Occlusion Pressure in Patients with and Without Pulmonary Hypertension. Chest.

[68] Nemoto K., Oh-Ishi S., Akiyama T., Yabuuchi Y., Goto H., Nonaka M., Sasatani Y., Tachi H., Arai N., Ishikawa H. (2019). Borderline pulmonary hypertension is associated with exercise intolerance and increased risk for acute exacerbation in patients with interstitial lung disease. BMC Pulm. Med..

[69] Simonneau G., Hoeper M.M. (2019). The revised definition of pulmonary hypertension: Exploring the impact on patient management. Eur. Heart J. Suppl..

[70] McLaughlin V.V., Shillington A., Rich S. (2002). Survival in Primary Pulmonary Hypertension. Circulation.

[71] Brusca S.B., Zou Y., Elinoff J.M. (2020). How low should we go? Potential benefits and ramifications of the pulmonary hypertension hemodynamic definitions proposed by the 6th World Symposium. Curr. Opin. Pulm. Med..

[72] Kempton H., Hungerford S., Muller D.W., Hayward C.S. (2024). Pulmonary arterial compliance as a measure of right ventricular loading in mitral regurgitation. IJC Heart Vasc..

[73] Bech-Hanssen O., Lindow T., Astengo M., Bollano E., Ricksten S.E. (2024). Echocardiographic Grading of Right Ventricular Afterload in Left Heart Disease: Relation to Right Ventricular Function, Pulsatile and Resistant Load, and Outcome. Pulm. Circ..

[74] Finkelhor R.S., Lewis S.A., Pillai D. (2015). Limitations and Strengths of Doppler/Echo Pulmonary Artery Systolic Pressure–Right Heart Catheterization Correlations: A Systematic Literature Review. Echocardiography.

[75] Bach D.S., Curtis J.L., Christensen P.J., Iannettoni M.D., Whyte R.I., Kazerooni E.A., Armstrong W., Martinez F.J. (1998). Preoperative Echocardiographic Evaluation of Patients Referred for Lung Volume Reduction Surgery. Chest.

[76] Rich J.D., Shah S.J., Swamy R.S., Kamp A., Rich S. (2011). Inaccuracy of Doppler Echocardiographic Estimates of Pulmonary Artery Pressures in Patients with Pulmonary Hypertension: Implications for Clinical Practice. Chest.

[77] Sonaglioni A., Cassandro R., Luisi F., Ferrante D., Nicolosi G.L., Lombardo M., Anzà C., Harari S. (2021). Correlation Between Doppler Echocardiography and Right Heart Catheterisation-Derived Systolic and Mean Pulmonary Artery Pressures: Determinants of Discrepancies Between the Two Methods. Heart Lung Circ..

[78] Rudski L.G., Gargani L., Armstrong W.F., Lancellotti P., Lester S.J., Grünig E., D’Alto M., Åström Aneq M., Ferrara F., Saggar R. (2018). Stressing the Cardiopulmonary Vascular System: The Role of Echocardiography. J. Am. Soc. Echocardiogr..

[79] Mukherjee M., Rudski L.G., Addetia K., Afilalo J., D’Alto M., Freed B.H., Friend L.B., Gargani L., Grapsa J., Hassoun P.M. (2025). Guidelines for the Echocardiographic Assessment of the Right Heart in Adults and Special Considerations in Pulmonary Hypertension: Recommendations from the American Society of Echocardiography. J. Am. Soc. Echocardiogr..

[80] Luong C.L., Anand V., Padang R., Oh J.K., Arruda-Olson A.M., Bird J.G., Pislaru C., Thaden J.J., Pislaru S.V., Pellikka P.A. (2024). Prognostic Significance of Elevated Left Ventricular Filling Pressures with Exercise: Insights from a Cohort of 14,338 Patients. J. Am. Soc. Echocardiogr..

[81] Dweik R.A., Rounds S., Erzurum S.C., Archer S., Fagan K., Hassoun P.M., Hill N.S., Humbert M., Kawut S.M., Krowka M. (2014). An Official American Thoracic Society Statement: Pulmonary Hypertension Phenotypes. Am. J. Respir. Crit. Care Med..

[82] Hemnes A.R., Beck G.J., Newman J.H., Abidov A., Aldred M.A., Barnard J., Berman Rosenzweig E., Borlaug B.A., Chung W.K., Comhair S.A.A. (2017). PVDOMICS. Circ. Res..

[83] Hemnes A.R., Leopold J.A., Radeva M.K., Beck G.J., Abidov A., Aldred M.A., Barnard J., Rosenzweig E.B., Borlaug B.A., Chung W.K. (2022). Clinical Characteristics and Transplant-Free Survival Across the Spectrum of Pulmonary Vascular Disease. J. Am. Coll. Cardiol..

[84] Jellis C.L., Park M.M., Abidov A., Borlaug B.A., Brittain E.L., Frantz R., Hassoun P.M., Horn E.M., Jaber W.A., Jiwon K. (2022). Comprehensive echocardiographic evaluation of the right heart in patients with pulmonary vascular diseases: The PVDOMICS experience. Eur. Heart J.—Cardiovasc. Imaging.

[85] Borlaug B.A., Larive B., Frantz R.P., Hassoun P., Hemnes A., Horn E., Leopold J., Rischard F., Berman-Rosenzweig E., Beck G. (2024). Pulmonary hypertension across the spectrum of left heart and lung disease. Eur. J. Heart Fail..

[86] Lam C.S.P., Lyass A., Kraigher-Krainer E., Massaro J.M., Lee D.S., Ho J.E., Levy D., Redfield M.M., Pieske B.M., Benjamin E.J. (2011). Cardiac Dysfunction and Noncardiac Dysfunction as Precursors of Heart Failure with Reduced and Preserved Ejection Fraction in the Community. Circulation.

[87] Shah S.J., Katz D.H., Selvaraj S., Burke M.A., Yancy C.W., Gheorghiade M., Bonow R.O., Huang C.-C., Deo R.C. (2015). Phenomapping for Novel Classification of Heart Failure with Preserved Ejection Fraction. Circulation.

[88] Harder E.M., Divo M.J., Washko G.R., Leopold J.A., Rahaghi F.N., Waxman A.B. (2024). Implications of Mean Pulmonary Arterial Wedge Pressure Trajectories in Pulmonary Arterial Hypertension. Am. J. Respir. Crit. Care Med..

[89] McLaughlin V.V., Vachiery J.-L., Oudiz R.J., Rosenkranz S., Galiè N., Barberà J.A., Frost A.E., Ghofrani H.-A., Peacock A.J., Simonneau G. (2019). Patients with pulmonary arterial hypertension with and without cardiovascular risk factors: Results from the AMBITION trial. J. Heart Lung Transplant..

[90] Reddy Y.N.V., Frantz R.P., Hassoun P.M., Hemnes A.R., Horn E., Leopold J.A., Rischard F., Rosenzweig E.B., Hill N.S., Erzurum S.C. (2024). Clinical Implications of Pretest Probability of HFpEF on Outcomes in Precapillary Pulmonary Hypertension. J. Am. Coll. Cardiol..

[91] Omote K., Verbrugge F.H., Sorimachi H., Omar M., Popovic D., Obokata M., Reddy Y.N.V., Borlaug B.A. (2023). Central haemodynamic abnormalities and outcome in patients with unexplained dyspnoea. Eur. J. Heart Fail..

[92] Opitz C.F., Hoeper M.M., Gibbs J.S.R., Kaemmerer H., Pepke-Zaba J., Coghlan J.G., Scelsi L., D’Alto M., Olsson K.M., Ulrich S. (2016). Pre-Capillary, Combined, and Post-Capillary Pulmonary Hypertension: A Pathophysiological Continuum. J. Am. Coll. Cardiol..

[93] Mak S., Kolker S., Girdharry N.R., Bentley R.F., Valle F.H., Gurtu V., Mok K.H., Moric J., Thenganatt J., Granton J.T. (2022). The role of exercise right heart catheterization to guide pulmonary hypertension therapy in older adults. Pulm. Circ..

[94] Ameri P., Mercurio V., Pollesello P., Anker M.S., Backs J., Bayes-Genis A., Borlaug B.A., Burkhoff D., Caravita S., Chan S.Y. (2024). A roadmap for therapeutic discovery in pulmonary hypertension associated with left heart failure. A scientific statement of the Heart Failure Association (HFA) of the ESC and the ESC Working Group on Pulmonary Circulation. Eur. J. Heart Fail..

[95] Borlaug B.A., Reddy Y.N.V., Braun A., Sorimachi H., Omar M., Popovic D., Alogna A., Jensen M.D., Carter R. (2023). Cardiac and Metabolic Effects of Dapagliflozin in Heart Failure with Preserved Ejection Fraction: The CAMEO-DAPA Trial. Circulation.

[96] Kayano H., Koba S., Hirano T., Matsui T., Fukuoka H., Tsuijita H., Tsukamoto S., Hayashi T., Toshida T., Watanabe N. (2020). Dapagliflozin Influences Ventricular Hemodynamics and Exercise-Induced Pulmonary Hypertension in Type 2 Diabetes Patients ―A Randomized Controlled Trial―. Circ. J..

[97] Nassif M.E., Qintar M., Windsor S.L., Jermyn R., Shavelle D.M., Tang F., Lamba S., Bhatt K., Brush J., Civitello A. (2021). Empagliflozin Effects on Pulmonary Artery Pressure in Patients with Heart Failure. Circulation.

[98] Ge T., Yang Y., Zhao Y. (2022). A study of the efficacy of sacubitril/valsartan plus dapagliflozin combination treatment in pulmonary arterial hypertension due to left heart disease. Perfusion.

[99] Codina P., Domingo M., Barceló E., Gastelurrutia P., Casquete D., Vila J., Abdul-Jawad Altisent O., Spitaleri G., Cediel G., Santiago-Vacas E. (2022). Sacubitril/valsartan affects pulmonary arterial pressure in heart failure with preserved ejection fraction and pulmonary hypertension. ESC Heart Fail..

[100] McMurray J.J.V., Jackson A.M., Lam C.S.P., Redfield M.M., Anand I.S., Ge J., Lefkowitz M.P., Maggioni A.P., Martinez F., Packer M. (2020). Effects of Sacubitril-Valsartan Versus Valsartan in Women Compared with Men with Heart Failure and Preserved Ejection Fraction. Circulation.

[101] Shah S.J., Bonderman D., Borlaug B.A., Cleland J.G.F., Lack G., Lu W., Voors A.A., Zannad F., Gladwin M.T. (2025). Macitentan for Heart Failure with Preserved or Mildly Reduced Ejection Fraction and Pulmonary Vascular Disease: Results of the SERENADE Randomized Clinical Trial and Open-Label Extension Study. Circ. Heart Fail..

[102] Cooper T.J., Cleland J.G.F., Guazzi M., Pellicori P., Ben Gal T., Amir O., Al-Mohammad A., Clark A.L., McConnachie A., Steine K. (2022). Effects of sildenafil on symptoms and exercise capacity for heart failure with reduced ejection fraction and pulmonary hypertension (the SilHF study): A randomized placebo-controlled multicentre trial. Eur. J. Heart Fail..

[103] Burkhoff D., Borlaug B.A., Shah S.J., Zolty R., Tedford R.J., Thenappan T., Zamanian R.T., Mazurek J.A., Rich J.D., Simon M.A. (2021). Levosimendan Improves Hemodynamics and Exercise Tolerance in PH-HFpEF: Results of the Randomized Placebo-Controlled HELP Trial. JACC Heart Fail..

[104] Armstrong P.W., Pieske B., Anstrom K.J., Ezekowitz J., Hernandez A.F., Butler J., Lam C.S.P., Ponikowski P., Voors A.A., Jia G. (2020). Vericiguat in Patients with Heart Failure and Reduced Ejection Fraction. N. Engl. J. Med..

[105] Udelson J.E., Lewis G.D., Shah S.J., Zile M.R., Redfield M.M., Burnett J., Parker J., Seferovic J.P., Wilson P., Mittleman R.S. (2020). Effect of Praliciguat on Peak Rate of Oxygen Consumption in Patients with Heart Failure with Preserved Ejection Fraction: The CAPACITY HFpEF Randomized Clinical Trial. JAMA.

[106] Zhang H., Zhang J., Chen M., Xie D.-J., Kan J., Yu W., Li X.-B., Xu T., Gu Y., Dong J. (2019). Pulmonary Artery Denervation Significantly Increases 6-Min Walk Distance for Patients with Combined Pre- and Post-Capillary Pulmonary Hypertension Associated with Left Heart Failure: The PADN-5 Study. JACC Cardiovasc. Interv..

[107] Vachiéry J.-L., Delcroix M., Al-Hiti H., Efficace M., Hutyra M., Lack G., Papadakis K., Rubin L.J. (2018). Macitentan in pulmonary hypertension due to left ventricular dysfunction. Eur. Respir. J..

[108] Koller B., Steringer-Mascherbauer R., Ebner C.H., Weber T., Ammer M., Eichinger J., Pretsch I., Herold M., Schwaiger J., Ulmer H. (2017). Pilot Study of Endothelin Receptor Blockade in Heart Failure with Diastolic Dysfunction and Pulmonary Hypertension (BADDHY-Trial). Heart Lung Circ..

[109] Hoendermis E.S., Liu L.C.Y., Hummel Y.M., Van Der Meer P., De Boer R.A., Berger R.M.F., Van Veldhuisen D.J., Voors A.A. (2015). Effects of sildenafil on invasive haemodynamics and exercise capacity in heart failure patients with preserved ejection fraction and pulmonary hypertension: A randomized controlled trial. Eur. Heart J..

[110] Bonderman D., Ghio S., Felix S.B., Ghofrani H.-A., Michelakis E., Mitrovic V., Oudiz R.J., Boateng F., Scalise A.-V., Roessig L. (2013). Riociguat for Patients with Pulmonary Hypertension Caused by Systolic Left Ventricular Dysfunction. Circulation.

[111] Lewis G.D., Shah R., Shahzad K., Camuso J.M., Pappagianopoulos P.P., Hung J., Tawakol A., Gerszten R.E., Systrom D.M., Bloch K.D. (2007). Sildenafil Improves Exercise Capacity and Quality of Life in Patients with Systolic Heart Failure and Secondary Pulmonary Hypertension. Circulation.

[112] Califf R.M., Adams K.F., McKenna W.J., Gheorghiade M., Uretsky B.F., McNulty S.E., Darius H., Schulman K., Zannad F., Handberg-Thurmond E. (1997). A randomized controlled trial of epoprostenol therapy for severe congestive heart failure: The Flolan International Randomized Survival Trial (FIRST). Am. Heart J..

[113] Gerges C., Gerges M., Lang M.B., Zhang Y., Jakowitsch J., Probst P., Maurer G., Lang I.M. (2013). Diastolic Pulmonary Vascular Pressure Gradient: A Predictor of Prognosis in “Out-of-Proportion” Pulmonary Hypertension. Chest.

[114] Zotter-Tufaro C., Duca F., Kammerlander A.A., Koell B., Aschauer S., Dalos D., Mascherbauer J., Bonderman D. (2015). Diastolic Pressure Gradient Predicts Outcome in Patients with Heart Failure and Preserved Ejection Fraction. J. Am. Coll. Cardiol..

[115] Tampakakis E., Leary P.J., Selby V.N., De Marco T., Cappola T.P., Felker G.M., Russell S.D., Kasper E.K., Tedford R.J. (2015). The Diastolic Pulmonary Gradient Does Not Predict Survival in Patients with Pulmonary Hypertension Due to Left Heart Disease. JACC Heart Fail..

[116] Brittain E.L., Assad T.R., Hemnes A.R., Newman J.H. (2015). The Diastolic Pressure Gradient Does Not—And Should Not—Predict Outcomes. JACC Heart Fail..

[117] Aronson D., Eitan A., Dragu R., Burger A.J. (2011). Relationship Between Reactive Pulmonary Hypertension and Mortality in Patients with Acute Decompensated Heart Failure. Circ. Heart Fail..

[118] Palazzini M., Dardi F., Manes A., Bacchi Reggiani M.L., Gotti E., Rinaldi A., Albini A., Monti E., Galiè N. (2018). Pulmonary hypertension due to left heart disease: Analysis of survival according to the haemodynamic classification of the 2015 ESC/ERS guidelines and insights for future changes. Eur. J. Heart Fail..

[119] Vanderpool R.R., Saul M., Nouraie M., Gladwin M.T., Simon M.A. (2018). Association Between Hemodynamic Markers of Pulmonary Hypertension and Outcomes in Heart Failure with Preserved Ejection Fraction. JAMA Cardiol..

[120] Pellegrini P., Rossi A., Pasotti M., Raineri C., Cicoira M., Bonapace S., Dini F.L., Temporelli P.L., Vassanelli C., Vanderpool R. (2014). Prognostic Relevance of Pulmonary Arterial Compliance in Patients with Chronic Heart Failure. Chest.

[121] Al-Naamani N., Preston I.R., Paulus J.K., Hill N.S., Roberts K.E. (2015). Pulmonary Arterial Capacitance Is an Important Predictor of Mortality in Heart Failure with a Preserved Ejection Fraction. JACC Heart Fail..

[122] Mohammed S.F., Hussain I., Abouezzeddine O.F., Takahama H., Kwon S.H., Forfia P., Roger V.L., Redfield M.M. (2014). Right Ventricular Function in Heart Failure with Preserved Ejection Fraction. Circulation.

[123] Pacher R., Stanek B., Hülsmann M., Koller-Strametz J., Berger R., Schuller M., Hartter E., Ogris E., Frey B., Heinz G. (1996). Prognostic impact of big endothelin-1 plasma concentrations compared with invasive hemodynamic evaluation in severe heart failure. J. Am. Coll. Cardiol..

